# Hydropathic and Other Establishments

**Published:** 1901-04-20

**Authors:** 


### HYDROPATHIC AND OTHER ESTABLISHMENTS.

Alexandra Hospital, Woodhall Spa, Lincolnshire.—Accommodation: 28 beds. Medical officers: Drs. C. J. Williams and H. W. Gwyn. Treatment: By baths and mineral waters. Nearest railway station, Woodhall Spa.

Approximate fees per week, 10s.

Ben Rhydding Hydropathic Establishment and Hotel, Yorkshire. — Situated at Wharfedale, Yorkshire. Accommodation: 120 bedrooms, 5 reception rooms. Medical officers: Dr. Thomas Scott, Consulting Physician; Dr. W. R. Bates, Physician. General hydropathic treatment. Nearest railway station, Ben Rhydding.

Approximate fees, from £2 12s. 6d. per week.

Blackpool Imperial Hydropathic Hotel, Claremont Park.—Situation: Looking out on the Irish Sea. 180 bed and sitting rooms. Physician attends the hotel daily. Russian and Turkish baths.

Approximate fees per week, £3 10s. to £4 4s.

Bournemouth Hydropathic, Bournemouth (West Cliff).—South aspect, overlooking sea; sheltered amongst pines. Accommodation: Beds, 60; reception rooms, 4. Medical officer: Dr. W. Johnson Smyth. Turkish, sea-water, pine, sulphur, and electric baths; massage. Communications to Lady Secretary. Nearest railway station, Bournemouth, West.

Approximate fees, £3 3s. per week.

Bridge of Allan Hydropathic, N.B.—South aspect. 100 beds, 4 public rooms. Medical officer: Dr. Archd. Mackintosh. Treatment: Hydropathic, including massage. Railway station, Bridge of Allan, N.B., Caledonian Railway.

Approximate fees per week, 2½ guineas to 3 guineas.

The Dowsing Institution, 28 York Place, Baker Street, W.—Branches: 8 Uxbridge Road, Ealing, W., 18 Crossfield Road, Swiss Cottage, N.W., 258 Elgin Avenue, W., 17 Clifton Villas, Maida Hill, W., and at Belfast, Bexhill, Bradford, Bristol, Brighton, Buxton, Cheltenham, Croydon, Cardiff, Droitwich, Doncaster, Dublin, Edinburgh, Exeter, Greenock, Hastings, Hove, Harrogate, Hull, Kew Gardens, Leamington, Llandrindod, London, Manchester, Matlock, Newcastle, Norwich, Nottingham, Oxford, Plymouth, Scarborough, Southport, Sheffield, St. Leonards. Radiant heat and light bath and appliances, also electric, water, and other baths, Swedish gymnastics, Lupus treatment.

Glenburn Hydropathic Establishment, Rothesay, N.B.—Bedrooms, 125; reception rooms, 8. Medical Officer: Dr. Wm. C. Philp. Special features of treatment: Sea-water baths, Turkish, Russian, spray, douche, etc.; massage; Nauheim baths; electric light bath; electric baths; static electricity; high frequency treatment; electric vibration and massage. Nearest railway station, Rothesay, N.B.

Approximate fees, £3 3s. to £4 4s. per week.

Haddon Hall and Haddon Grove Hydros, London Road, Buxton.—Accommodation for 80. Treatment by electric baths, massage, &c. Nearest railway station, Buxton.

Approximate fees, £2 9s. to £3 3s. per week.

Kenworthy's Hydropathic, 51 to 57 Bath Street, Southport. 80 bedrooms. Medical officer: Dr. A. B. Kenworthy. Special features of treatment: Thorough hydro-therapeutic treatment. Dowsing radiant heat treatment. Turkish, Russian, vapour, douche, needle, spray, slipper, wave &c., baths; electro-chemical baths, massage. Nearest railway station, Chapel Street, Southport (L. & Y. Ry.). Best seasons for Southport, autumn, winter, and spring.

Approximate fees per week, from £2 2s. to £3 3s.

Matlock House Hydro, Matlock, Derbyshire.—In the Peak District, 700 feet above sea-level, with due south aspect. Accommodation for 70 persons. Medical officer: Dr. W. Moxon. Hydropathic baths of every description; electric, Nauheim and other treatment. Nearest railway station, Matlock Bridge.

Approximate fees, £2 2s. to £4 4s. per week.

Northcote Hydro, Woodhall Spa, Lincoln.—Accommodation, 30 beds. Medical officer: Dr. Robert Cuffe. Special features: Treatment by the bromo-iodine baths, electrotherapy. Nearest railway station, Woodhall Spa.

Royal West of England Sanatorium, Weston-super-Mare.—Sheltered by the Mendip Hills, and facing the Atlantic. Beds, 140. Visiting medical officer: Dr. Fraser. Special treatment: Warm sea-water baths. Communications to Weston-super-Mare. The Great Western Railway issue tickets to patients at reduced charges.

Approximate fees for a fortnight from March to October, 31s.; the rest of the year, 26s.

Shandon Hydropathic, Shandon, N.B.—On the shores of the Gareloch. 120 bedrooms. Medical officer: Dr. Douglas Reid. Treatment: Hydropathic, including Turkish, Russian, and sea-water baths. Nearest railway station, Shandon.

Smedley's Hydropathic Establishment, Matlock Bridge, Derbyshire.—Hill side, south-west, 500 feet above sea-level. Accommodation: Nearly 300 beds. Medical staff: Dr. W. Cecil Sharpe, senior physician, and Dr. George C. R. Harbinson, resident physician. Special features: Turkish, Russian, electric, radiant, heat baths, air douche, massage, and electricity; Weir-Mitchell and Nauheim treatment, &c. Nearest railway station, Matlock Bridge.

Approximate fees per week, 3 to 5 guineas.

The Tallerman Institute, 50 Welbeck Street, W.—Local application of superheated dry air. Arrangements can be made for the treatment of patients by the Tallerman apparatus, under medical direction, either at the Institute, or at their own homes.

